# Why does obesity promote cancer? Epidemiology, biology, and open questions

**DOI:** 10.3332/ecancer.2015.554

**Published:** 2015-07-23

**Authors:** Luca Mazzarella

**Affiliations:** European Institute of Oncology, Via Ripamonti 435, Milan 20141, Italy

**Keywords:** obesity, body mass index, insulin, inflammation, DNA damage

## Abstract

The association between obesity and/or metabolic syndrome and an elevated mortality from cancer has been confirmed by an astonishing number of studies across nations and ethnicities, such that obesity is now recognised to be among the most prominent cancer risk factors worldwide.

Despite this overwhelming evidence and the societal impact of obesity, we know surprisingly little about the underlying molecular mechanisms. This knowledge gap is a major obstacle to the implementation of effective lifestyle change policies. As the scientific community is insecure on what messages it should deliver, administrators are uncertain as to what exactly to recommend, and consumers are confused about whom to believe. This leaves the field flooded with pseudo-scientific recommendations that are hard to eradicate.

In this review, I will provide a summary of the existing epidemiological and mechanistic evidence on the relationship between systemic metabolism and cancer, highlighting debated issues and ongoing investigations.

## Introduction

The world has become a fatter place to live in the last few decades. Western countries now have a significant fraction of their inhabitants who are overweight or obese. In the US, this has reached apocalyptic proportions, with 30% of the adult population overweight and an astonishing 35% being obese [1]. In the UK, where the prevalence of obesity is among the highest in Europe, obesity is considered to be the second most important avoidable risk factor for cancer after smoking [2]. The rest of the world, especially the rising-economic countries, is doing its best to catch up. A recent study estimated that the contribution of overweight/obesity to cancer risk (measured as population attributable fraction) in areas with the largest increases in BMI in the last two decades, like Latin America and the Caribbean and the Middle-East (2.0–9.9%), has now reached levels observed in Northern America (3.5–9.4%) [3].

A fatter world means an elevated incidence of chronic diseases, with cancer playing a leading role. Obese people are much more likely to die of cancer than non-obese people, as evidenced by large prospective trials that we review here. The reasons for this association between obesity and cancer death are not immediately understood.

### A historical perspective

The first evidence on the relationship between obesity and/or metabolic dysfunction and cancer dates back to the 50s for experimental models and to the 60s for epidemiological studies. Although we now know that other cancers show stronger dependency on obesity, much of the initial work investigated is on the breast cancer. Authors at Stanford showed that if obesity was induced experimentally by chemically ablating the hypothalamic appetite control, the spontaneous incidence of tumours (especially breast) increased dramatically in inbred mouse strains with inherent cancer susceptibility [4, 5]. In the same period, it was shown that surgical castration greatly diminished disease penetrance and delayed latency in experimental models of breast cancer [5], suggesting a fundamental role for oestrogens. Thus, it appeared very likely from the beginning that obesity may influence breast cancer risk by modulating oestrogen levels.

It took more or less another decade before the laboratory evidence translated into clinical evidence. Landmark studies were published in 1964 by a Dutch group [6]. It had already been known for a long time that breast cancer follows a bimodal incidence distribution, peaking before and after menopause. The cause of such bimodality puzzled many researchers of the time, but it was fairly established that it must be because of alterations in oestrogen physiology, as oestrogens were known to be produced by the ovaries in premenopause and by the adrenal glands in postmenopause. Using a remarkable combination of biochemistry and epidemiology (what would today be called ‘molecular epidemiology’), the group showed that obese postmenopausal women maintained signs of elevated oestrogen activity despite loss of ovarian secretion. Obesity or glucose intolerance was associated only with breast cancer in postmenopause (when obesity correlated with higher oestrogens), but not in premenopause (when obesity did not influence oestrogen levels). A long-lasting paradigm was born: obesity increases the incidence of postmenopausal breast cancer by causing persistently elevated adrenal oestrogen levels. Confirming this paradigm, castrated mice still showed increased breast cancer susceptibility if rendered obese [5].

In many ways these early studies were key to forming the ideas that occupy the debate in the present day too. First, the idea that circulating factors (like oestrogens) do not simply correlate with risk but are causative and represent good biomarkers to evaluate risk. Second, the idea that tumours arising in different biological contexts (eg. premenopausal versus postmenopausal breast cancer) respond in different ways to obesity. Third, the idea that it is metabolic dysfunction (as revealed by glucose intolerance) and not obesity per se (as simple increase in body size) that augments cancer risk.

## The size of the problem

With the publication of several large prospective cohort studies in the last 15 years [7–13], the strength of the association between obesity and cancer became evident (Table 1).

All studies consistently found increased hazard ratios (HR) of developing tumours in most anatomical sites for overweight/obese subjects. Exceptions are lung, brain, and melanoma, where presumably the influence of other environmental factors—smoke or ultraviolet (UV)— is dominant, or where the target tissue is anatomically shielded from metabolic fluctuations (brain).

On the other hand, endometrial, kidney, and hepatobiliary cancers are the most strongly affected in the majority of studies.

An early influential study in the field was the analysis of the Cancer Prevention Study II, which enroled and followed up more than one million Americans of disparate ethnicities and in which anthropometric data were assessed by a mailed questionnaire [8]. In the first analysis, authors analysed cancer mortality (an easier parameter to score in national databases), obtaining very elevated HR (up to >6), much higher than subsequent studies assessing incidence. In retrospect, this magnitude difference is instructive, as we now know that obesity is also associated with poorer outcome after cancer diagnosis. Thus, obese subjects are at a higher risk of getting cancer, and once diagnosed, of dying from it. This is important to highlight as the mechanisms underlying risk or outcome may differ considerably and may suggest different lifestyle policies depending on whether the aim is to *prevent* or *treat* cancer.

We will now briefly review the epidemiological evidence relating to the most epidemiologically relevant anatomical sites.

### Breast cancer

The relationship between breast cancer and metabolic dysfunction is probably the most studied, and the most complex. Menopause represents a clear divide: risk of developing the disease in obese women is consistently increased in postmenopause, but just as consistently *decreased* in premenopause. The reasons for this paradox are still unclear. An early report by Potischman *et al* found evidence of an inverse relationship between BMI and serum total oestrogen levels in premenopause. This was speculated to be because of more frequent anovulatory cycles in obese women [21]. Although attractive, this model has not been replicated, and somehow confuted by a recent meta-analysis that found a *direct* relationship between free (bioavailable) oestradiol and BMI [22].

The clear role of oestrogens on postmenopausal breast cancer leads to speculate that only oestrogen-responsive breast tumours (as marked by their expression of oestrogen receptor, ER) are increased in obese postmenopausal women. Indeed this was confirmed by a meta-analysis by Suzuki *et al,* that showed that only ER+ breast cancers are increased in postmenopause (and, again decreased in premenopause), whereas ER-cancers seem unaffected by obesity [20]. However, a recent re-evaluation of the European Prospective Investigation into Cancer and Nutrtion (EPIC) cohort found that hormone replacement therapy (HRT) in the perimenopausal period, once common practice, might have confounded interpretation: if users of HRT in EPIC were omitted from the analysis, an increased HR in postmenopausal women was revealed also for ER-tumours (HR 1.59 for the highest versus the lowest BMI tertile) [12]. This might be taken as evidence for alternative, non-oestrogen-centric mechanisms for obesity effect on breast cancer.

The effect of obesity on breast cancer outcome is perhaps even stronger. In a nationwide study within the Danish Registry, in which almost 20.000 women treated for early breast cancer between 1977 and 2006 were analysed, the cumulative risk of relapsing by distant metastasis at ten years postdiagnosis was 46% higher in obese women, and the risk of dying of breast cancer after 30 years was higher by 38% [23]. A recent meta-analysis [18], pooling data from 82 studies and >200,000 patients (with no biological subgroup stratification), showed a consistently increased risk of breast cancer-specific mortality (between 25% and 68% higher, comparing obese with normal weight), whether the BMI was recorded before or after cancer diagnosis.

This picture becomes more complicated as we start to consider the interaction with specific biological subtypes and specific therapies. In two large adjuvant studies in patients with ER-positive disease, obesity was found to specifically reduce the advantage obtained by aromatase inhibitors (but not of tamoxifen), in both premenopausal (the ABSCG-12 trial [24]) and postmenopausal (the ATAC trial [25]). An intriguing mechanism was recently proposed to account for this effect (see section on oestrogen biology below). In our retrospective analysis of >1200 patients with HER-2+ breast cancer operated at our institution prior to the introduction of adjuvant trastuzumab, we observed a significant difference in disease free-survival (DFS) only for postmenopausal ER-patients, but not in premenopausal or ER+ patients. The most common event for relapse was distant metastases, which occurred about two fold more frequently in obese postmenopausal ER– patients [26].

Characteristically, distant metastases are associated with obesity, but not local recurrences. This suggests a true increased biological aggressiveness and not merely an increased likelihood of surgical inadequacy in the context of enlarged mammary tissue. As our understanding of this disease becomes more nuanced and treatment strategies more variegated, additional research on metabolic influences on outcome is warranted.

### Colorectal Cancer

Obesity moderately increases the risk of developing colorectal cancer, but this effect is consistently observed across several studies, and is higher in men [11, 27–29]. Interestingly, the strength of the association is higher with colic than with rectal localisation [11]. On the other hand, the effect of obesity on colorectal cancer outcome is still a matter of some controversy, as differences in data collection and the statistical model chosen result in different interpretations. Two recent meta-analyses came to conflicting results: Parkin *et al*, assuming a linear dose-response model, concluded that BMI increases did not significantly alter outcome [30]. However, this approach was criticised by Wu *et al*, who found that only obese (but not overweight) patients had a worse outcome; a possible reason for heterogeneity in study results was the timing of BMI assessment: if too close to the time of surgery, the strength of the relationship tended to weaken [31].

### Gynaecological cancers

Endometrial cancer is perhaps the disease in which obesity most strongly impacts survival. Risk of developing endometrial cancer is consistently increased several fold in all studies, with HRs up to >4 for obese versus normal weight and an average HR of 1.6 every 5 kg/m^2^ increase in a meta-analysis in 2010 (ref [32]). The relationship between metabolic dysfunction and endometrial cancer was also one of the first to be identified in the 60s [33]. The reasons for such a strong association are not entirely clear. Several possible mechanisms have been proposed, but epidemiological studies have so far failed to support a particular one: in a recent assessment of the Women’s Health Initiative, BMI was still a stronger independent risk factor than diabetes or HRT for the development of endometrial cancer [34].

The relationship between obesity and endometrial cancer recurrence and mortality is less clear, as few studies have directly assessed the issue. A meta-analysis in 2013 concluded that although overall mortality was increased with HRs ranging between 1.86–2.76, death specifically because of endometrial cancer was not significantly increased [35]. However, the same authors subsequently published results from their analysis on two large prospective cohorts (the NIH-AARP and the Women’s Health Initiative), in which the association between obesity and endometrial cancer-related mortality was statistically significant [36, 37].

### Liver cancers

Metabolic syndrome with one or more of its components (diabetes, obesity, and non-alcoholic fatty liver disease (NAFLD) is a major risk factor for development of hepatocellular carcinoma (HCC), especially in areas where chronic viral hepatitis has lower prevalence. This association is consistently stronger for males than for females and is conserved across ethnicities [38].

Interestingly, the way from metabolic syndrome/NAFLD to HCC does not necessarily require a cirrhotic stage, as suggested by several case reports and small series (reviewed in [39, 40]). Systemic and local inflammation seems to play a particularly strong role in metabolic syndrome-associated HCC (see section on inflammation below).

Little data is available on outcome: HCC arising in the context of NAFLD and obesity seems to have a worse prognosis. However, these tumours were already larger at diagnosis in a recent Australian series, which might in turn be because of relatively lower screening rates compared to cirrhotic patients [40].

## General issues in interpreting epidemiological studies on obesity-cancer relationship

Several millions of individuals have contributed data to studies addressing metabolism-cancer relationships, constituting probably the largest epidemiological cohort in history. However, pooling of data is made difficult by heterogeneity in the methodology of data collection and data analysis.

### BMI versus other anthropometric parameters

The use of BMI dates back to the 19th century and has become extremely popular as a means to evaluate a subject’s adiposity, thanks to its immediate definition as BMI=weight(kg)/(height(m)^2^) (which in itself is a further simplification. Its inventor, Quetelet, actually suggested a higher exponent than 2 to correct for disproportionate impact of weight as height increases). However its appropriateness to describe a subject’s adiposity has been questioned: BMI is particularly inaccurate at extreme heights (taller patients have inappropriately high values), to describe elderly population (who become shorter as an effect of ageing), and is poorly sensitive at identifying fat subjects within the normal-overweight BMI range. The implications were powerfully illustrated in a paper analysing the relationship between body fat percentage and BMI within the National Health and Nutrition Examination Survey (NHANES) study. Using BMI as a marker of obesity >50% of subjects with excess body fat would be classified as being normal or just overweight [14].

### BMI categorisation

The vast majority of studies tend to group individuals into BMI classes, usually on the basis of the very common World Health Organisation (WHO) classification into underweight (BMI<18.5), normal weight (BMI between 18.5 and 25), overweight (BMI between 25 and 30), and obesity (BMI>30). Although useful to immediately translate findings in clinical practice and to facilitate risk calculation, this imposes an artificial categorisation on what is really a continuous rather than discrete variable. It is worth recalling that the WHO classification is not based specifically on studies addressing cancer risk, but on a variety of evidence in which cardiovascular diseases play a key role [15].

When BMI is considered as a continuous variable, the shape of its association with cancer risk or outcome is often non-linear. Most commonly the relationship is J-shaped, with a disproportionately increased effect in obese versus overweight subjects [16–18]. In other cases, the relationship is U-shaped, suggesting that increased risk is also associated with low BMI, or can be sigmoidal, with increased risks in overweight subjects that do not further increase (or even decrease) in extremely obese subjects [7].

### Self-reported versus objectively collected parameters

As can be expected, body weight tends to be systematically underestimated when it is self-reported compared to an objective measurement conducted in the clinic. Of course, this is likely to introduce systematic errors in large epidemiological studies, especially when BMI is considered as a categorical value because large proportions of subjects can be inappropriately assigned to lower categories and thus significantly impair the power of statistical analysis.

A recent review tried to quantify this bias, analysing several studies where self-reported and objectively collected parameters could be directly compared (the analysis included also non-cancer related studies) [19]. The author concluded that BMI gets misclassified in 8.4–16.6% of cases overall, and obesity in particular in 4.4–11.9% of cases. Based on this review, one can safely conclude that the vast majority of the studies in which self-reported BMI is employed are likely to have provided reliable results with an error margin in the order of 10%. Some marginally significant trends may be interpreted with more confidence if such systematic error is taken into consideration

## What exactly is wrong with all this fat?

This is the crucial point that the medical community still lacks to this year of 2015: a unifying theory of how, in molecular terms, a systemic alteration in metabolism influences an individual’s chances of dying from cancer. It is unlikely that obesity per se is a strong cancer-promoting factor. Not all obese subjects develop cancer, and the relationship is rarely linear with increasing BMI. Furthermore, there are forms of experimentally induced obesity in which body size becomes more than six times the normal weight but no overt metabolic syndrome nor increased susceptibility to cancer is found [41]. Thus, obesity is likely to be the mirror of underlying dysfunctions, and only specific interaction with the genetic background will ultimately lead to increased susceptibility.

We will here attempt to reduce the possible mechanisms that link systemic metabolism with risk of dying from cancer into four broad categories: 1) a classic *initiating* carcinogenesis mechanism, because of accelerated accumulation of genomic alterations; 2) a classic *promotion* mechanism through alterations in the availability of nutrients and growth factors that are particularly important for tumour growth *after* its onset; 3) other biological mechanisms where both promotion and initiation mechanisms can be identified; 4) interference with therapeutic efficacy.

### 1. Obesity/metabolic syndrome as a cancer initiator

DNA accumulates mutations mostly through oxidative stress [42, 43]. This is brought about by excessive levels of oxygen radicals, either through excessive production (because of metabolic alterations or because of exogenous oxidative stimuli like alkylating agents or radiation), or defective elimination (because of antioxidant depletion). Oxygen radicals can also be produced by neighbouring cells as part of pathophysiological responses, most notably during inflammation (see relative section). Oxygen radicals are short-lived but exert part of their damaging activity by stably modifying longer-lived molecules. DNA is susceptible to oxidative attack either directly from reactive oxygen species (ROS) or indirectly through long-lived reactive species, most notably the lipid peroxidation products malondialdehyde and 4-hydroxynonenal, that are highly reactive but still longlived [44]. These processes lead to a wide range of covalent modifications of DNA, of variable mutagenic properties. Among the best studied are 8-oxo-dG, resulting from direct ROS attack, and the lipid peroxidation-derived M1-dG. Both have been demonstrated to be highly mutagenic in eukaryotic cells [45], but only few studies have directly assessed whether these molecules are actually accumulated in somatic cells and in obese individuals or experimental animals, usually finding positive correlations [46–48]. Most studies have focused on urinary or serum levels of M1-dG as a marker of oxidative stress, but its derivatives, more chemically stable and possibly more representative of long term exposure, have not been sufficiently studied for lack of reagents [49]. No study to date has unequivocally demonstrated that this mechanism is responsible for increased mutation accumulation *in vivo* in obese or metabolically impaired individuals.

Perhaps a so far neglected factor is the role of tissue stem cells. An established paradigm proposes that the cell of origin of cancer needs to live long enough and replicate sufficient times to accumulate the required set of DNA alterations (DNA mutations require replication for their fixation) [50]. The only cell types that continue replicating for the entire life span of an individual are adult stem cells (memory T cells are an exception, but recent research shows that these cells too show features of stem cells [51]). A highly controversial recent paper has indeed shown that the probability of getting cancer in a certain tissue is directly proportional to the total lifetime number of stem cell replication [52]. If we accept Vogelstein’s model, we must also conclude that we need to specifically study the effect of environmental factors on stem cells and neglect those on their short-lived progeny. In the specific case of metabolism, the issue is of particular importance: stem cells exhibit preference for specific metabolic pathways [53, 54], and this in turn may predispose them to peculiar types of metabolic damage. Stem cells comprise a tiny minority of the total cell population in every tissue, thus their associated DNA damage might have gone undetected in most studies to date.

### 2. Obesity/metabolic syndrome as a cancer promoter

In classical carcinogenesis, a *promoter* favours cancer by promoting the growth of the mutated cell where the mutation has been induced by another factor. Most of the existing literature on obesity and cancer has focused on this class of phenomena (for recent reviews, see [55, 56]). Individual mechanisms are likely to apply more specifically to certain cancer types.

#### Oestrogen biology in the obese environment

The striking association between female cancers (breast, endometrium) and obesity suggested early on that alterations in the homeostasis of female sex steroids play an important role. As mentioned above, aromatisation of adrenal gland-derived oestrogen precursors (androstenedione and testosterone) by P450 aromatase in the adipose tissue is increased in obese postmenopausal women. This mechanism is now well established and has been extensively reviewed elsewhere [55, 57].

The regulation of fat-associated aromatase expression is interesting from a molecular point of view. Tissue-specific isoforms arising from alternative promoter usage have been identified [58, 59]. Intriguingly, the fat-specific promoter is regulated by Jak/STAT upon activation by TNFa or IL6, suggesting a direct participation in inflammatory responses. The evolutionary advantage of such link is not of immediate understanding. In turn, exposure of breast preadypocytes to PPAR agonists led to decreased expression, suggesting a potential avenue for chemoprevention. Inflammation-dependent peripheral induction of aromatase activity [60] has been proposed to explain the impaired efficacy of aromatase inhibitors in obese patients highlighted and observed in the ATAC and ABSCG-12 trials [24, 25].

#### Insulin/IGF1, cancer and the diabetes connection

Insulin and the closely related Insulin-like Growth Factor 1 (IGF1) are well known hormones responsible for controlling the balance between catabolic and anabolic metabolism in most cells of the body. Their levels are modulated by the nutritional state through well-characterised feedback loops centred on the pancreas (for insulin) and the liver (for IGF1, under the control of growth hormone) (for a comprehensive review on insulin and IGF1 in cancer, see [61]). These loops are characteristically dysregulated in obese patients and in diabetes. Interestingly, in recent years additional levels of pathway modulation have been identified. Regarding insulin, several studies identified a role of inflammation in regulating peripheral sensitivity and glucose tolerance [62, 63]. Regarding IGF1, there is increasing realisation that paracrine (probably macrophage-derived) IGF1 plays at least as important a role as systemic (liver-derived) IGF1 in modulating tissue homeostasis and cancer. For instance, mice with a hepatocyte-specific loss of IGF1 expression were equally susceptible to IGF1-dependent cancerogenesis [64].

Insulin and IGF1 receptors share elevated sequence homology, and can heterodimerise with each other to form hybrid receptors. The importance of these receptors and mostly of IGF1R has long been recognised since the pivotal studies of Baserga and colleagues demonstrated that fibroblasts genetically lacking IGF1 receptor are resistant to tumoural transformation by a variety of classical oncogenes [65]. Many cancer cell lines express insulin and/or IGF1 and/or hybrid receptors, and depend on insulin/IGF1 for *in vitro* growth. However, all three types of receptor dimers can react with both insulin and IGF1, albeit with variable affinity, making it difficult to establish which ligand plays the main role in cancer. Some tumours produce their own autocrine IGF1 (not insulin), most notably myeloma [66], but the majority of tumours do not, and thus are likely to depend on systemic levels for their growth. Therefore, chronic alterations of insulin/IGF1 levels (or biological activity) because of systemic metabolic dysfunction like in obesity or diabetes are likely to create an environment in which cancer cells that depend on these factors thrive particularly well. Epidemiological evidence supporting an association between disruption in insulin/IGF1 homeostasis and cancer mortality is strong, but complicated by multiple confoundment [67]. Also very well known is the effect of caloric restriction (which dramatically reduces IGF1 and enhances insulin sensitivity) on reducing the incidence of spontaneous and chemically-induced tumours in rodents [68]. However, the beneficial effects of general caloric restriction appear to reduce as one climbs the evolutionary ladder, although with some caveats on the interpretation of data [69]. In primates, protein (especially of animal origin) rather than general caloric restriction significantly reduces circulating levels of IGF1. In a recent study in the NHANES cohort, however, this correlation held only at ages lower than 65. In older subjects, low protein consumption did not affect significantly IGF1 levels (which physiologically decrease with age) and were correlated with increased rather than decreased cancer mortality [70].

#### AMPK and the ongoing metformin ‘frenzy’

The involvement of the AMP-activated kinase AMPK has been the subject of extensive research in the last decade (reviewed in [71]), because of its central role in regulating the balance between anabolic and catabolic metabolism based on cellular energy state [72] and its involvement in cancer, either through direct alterations (rare) or loss of function of its regulator LKB1 (also known as STK11, long known to cause the hereditary Peutz-Jaeghers cancer syndrome) [73]. As alterations in AMPK function are observed in diabetic patients, this protein provided an interesting lead for the observed association between diabetes and cancer. The LKB1/AMPK axis was initially thought to be a direct target for the common anti-diabetic drug metformin [74], but this was found not to be the case in 2010 (ref [75]). Nevertheless, these findings provided the impetus for a flood of retrospective studies on the impact of metformin in cancer risk or outcome. Despite several conflicting results, meta-analyses concluded for an overall risk reduction of about 30% across all cancers, with peaks in pancreatic and liver cancers [76, 77]. Many studies may suffer from time-related biases (rate of metformin prescription increased over time, along with better therapy) [78]. Still, the initial enthusiasm for the possibility to repurpose a widely used drug for cancer treatment led to a second, and ongoing, flood of clinical trials testing the addition of metformin to standard therapy in a variety of tumours, either as a preventive or therapeutic strategy [79]. Currently, 114 open trials on metformin in cancer can be identified in Clinicaltrials.gov (retrieved on 12 March).

Some have questioned the design of most of these trials, because of the very wide inclusion criteria and the poor underlying molecular rationale [79]. Even in sensitive systems, the doses of metformin needed to achieve a cytotoxic effect are in the millimolar range *in vitro*, levels that are difficult to achieve *in vivo* without significant toxicity, even when potentially more active metformin analogues like phenformin are employed [79]. Furthermore, it is likely that only tumours with specific molecular alterations would realistically benefit from such treatments. The lack of a priori stratification might compromise the possibility of such trials to produce definitive evidence.

A further strategy to increase efficacy of metformin and its analogues may be through metabolic manipulation: dietary deprivation of serine was recently found to sensitise a variety of experimental tumours to the *in vivo* action of phenformin [80].

#### Other endocrine hormones and cancer

Other obesity-modulated circulating factors have been linked with cancer. Extensive evidence has been accumulated over the years for a direct promotion on tumour growth, usually through overexpression of the cognate receptor by tumour cells. The two best characterised factors are leptin [81] and endotrophin [82], whereas adiponectin, which is inversely correlated to body fat, has a less well characterised role. The interest in these factors is now mostly investigational rather than translational, as the opportunities to harness them for clinically useful scope seems little.

### 3. Other biological mechanisms

#### Inflammation: initiator, promoter, or neither?

Obesity is known to induce a state of chronic inflammation characterised by elevated cytokine levels and infiltration of innate and adaptive immune cells in organs devoted to metabolic control (adipose tissue, pancreas, and liver) [83]. Inflammation is considered by many to be at the crossroads of several pathogenetic mechanisms, characterised by a standardised response (in molecular terms, the usage of a restricted number of pathways) to noxious stimuli of infectious, and non-infectious nature. As cancer itself can cause tissue damage (through nutrient deprivation, cell death, ischemia, compression, etc.), it is often difficult to establish if inflammatory processes are cause or consequence of cancer initiation/promotion. Still, if inflammation plays a causative role, the final mechanism can be forced within the categories described in the previous sections, i.e. initiation (genotoxic activity) or promotion (imbalance of nutrient/growth factor signalling at the local or systemic level). These mechanisms include

- induction of oxidative stress in the surroundings of macrophage infiltration; this might contribute to DNA adduct formation as described above. This might trigger a self-reinforcing loop: several of the key inflammation mediators are ROS sensors themselves (like JNK and NfKB) [84]
- release of short-range cytokines to promote the local expansion of other immune cells and the regeneration of damaged tissue. These include strong inducers of proliferation, metastasis, and angiogenesis like the above-mentioned IGF1, PDGF, TGFb, FGF and VEGF, which have been found to be enriched in the mammary-associated fat in obese mice and humans [85]
- release of long-range cytokines (IL6, TNFa) [86] that may promote insulin resistance in the liver and other metabolism-controlling organs [62], thus generating increased levels of circulating insulin that may in turn promote cancer growth [85].

Inflammation is thought by many to be the missing link to explain how obesity promotes cancer. However, hardcore experimental evidence to clearly establish causation is difficult to obtain, given the complex interactions at play. In the setting of experimental HCC, landmark studies from the Karin’s lab identified IL6, NFkB, and STAT3-as crucial mediators, as genetic ablation of such factors in mice abrogated the tumourigenic effect of obesity [87, 88]. In other cancer models the evidence is less cogent; this certainly represents a very active line of research (see references 55 and 83 for extensive reviews on the topic).

#### The contribution of the microbiota

The study of the gut microbiota and its role in human disease is certainly one of the most rapidly developing fields of modern medicine. The notion that the flora composition is modulated by the diet appears obvious, but recent findings on how the flora itself can heavily influence physiology and pathology appear more and more striking as they come out. Particularly interesting for therapeutic scopes is the possibility to perform microbiota transplants (until lately only in animals, but recently also in humans) to modulate the host’s gut environment, and much more. Increase in body fat content can be induced into germ-free mouse by simply transplanting the gut flora of obese mice [89]. The human equivalent of this experiment was recently reported, as a woman receiving a fecal transplant from an overweight donor developed significant post-transplant weight gain [90]. Equally striking results have been recently obtained in studies on cancer initiation/promotion. A group from Germany reported that K-ras-dependent development of intestinal cancer in a mouse model was increased by high fat diet(HFD); intriguingly, the simple transplant of feces from HFD mice with tumours into tumour-free mice was sufficient to increase tumour development, and this could be blocked by antibiotic treatment or by oral supplementation with butyrate (a short-chain fatty acid previously demonstrated to modulate gut flora) [91].

Similar results were obtained by a Japanese group on carcinogen-induced liver cancers, which again arose more frequently in HFD-fed mice and could be reduced by antibiotic treatment (although no transplantability was assessed in this study) [92].

The possibility to control at least some of the environmental effects (including diet) on cancer by acting on the microbiota is obviously a very palatable alternative, likely to be much better tolerated than lifestyle intervention programmes. It will be interesting to find out whether the microbiota influence can extend also beyond the sites of immediate influence like gut and liver.

### 4. Just taking too little? Effect of obesity on pharmacological parameters

Reduced efficacy of chemotherapy and other treatments in overweight/obese patients is a likely and often underestimated factor contributing to increased cancer-related mortality. Decreased efficacy can occur through two main classes of mechanisms:

1. Modification of physiological parameters of the patient, leading to impaired pharmacokinetics. Glomerular filtration rate and liver enzyme function are both altered in obese individuals, although there is considerable debate over the best parameter to account for such modifications. Lean body weight seems to correlate linearly with drug clearance [93], but could not improve clearance prediction for most chemotherapeutic drugs evaluated in a further study [94]. As can be expected, data on pharmacokinetics in obese patients in randomised trials is very limited, mostly because these patients are systematically counter selected at enrolment. For an extensive review, see reference [95].
2. Inappropriate dose modification according to body weight. In regular practice, physicians often feel uneasy with administering extremely elevated doses of chemotherapeutics according to exact weight adjustments, for fear of excessive toxicity. The dose is often ‘capped’ either to alternative measures (e.g., ideal body weight) or in completely arbitrary ways. Given the close relationship between dose intensity and response rate for classical chemotherapeutic agents, this could in turn lead to reduced therapeutic effect. This has been demonstrated in retrospective analyses, especially in the setting of adjuvant therapy for breast cancer. For instance, in a retrospective analysis of four adjuvant trials led by the International Breast Cancer Study Group, a higher relapse rate and lower survival rate was observed in obese patients with oestrogen receptor–negative breast cancer who received < 85% of the dose [96]. A recent French retrospective analysis on a large (5000) population of early stage breast cancer patients treated with adjuvant anthracyclines ± taxanes concluded that when therapy was administered at the right, actual body surface-calculated density, there was no detrimental effect of BMI on outcome [97]. Similar findings were observed in the neoadjuvant setting, in the GeparQuattro study [98]. More research would be required to precisely quantify the impact of dose reduction in other settings.

To attempt to base clinical practice on evidence, ASCO recently extensively reviewed the literature and issued guidelines for dosing in overweight/obese patients [99]. The review found insufficient evidence for increased toxicity in obese patients receiving chemotherapy dosed on actual weight, and recommended that no dose correction be applied, especially when the goal of treatment is cure.

## Is obesity really a modifiable risk factor?

Obesity is clearly a modifiable trait, but what is still insufficiently grounded in evidence is the possibility for any weight reduction programme, however achieved, to really *decrease the risk of cancer death*. As we saw above, in fact, the underlying molecular mechanism gives rise to radically different scenarios:

1. Obesity may act mainly by modulating the biological activity of cancer cell- extrinsic factors, whose activity is immediate and limited in time (cytokines, hormones, nutrients, immunity, tumour microenvironment, etc.). If this scenario is true, lifestyle interventions can be effective inasmuch as they are able to modify these mediators. Since it is unlikely that all factors can be equally modulated by the same intervention, it will be important to characterise the interaction between each factor and lifestyle so that targets and biomarkers can be identified.
2. Obesity may also act by modulating cancer or precancerous cell-intrinsic factors, like genomic stability, clonal selection, epigenetic factors, or stem cell self renewal. In this case, lifestyle modifications can still be effective, but they may be predicted to have a bigger impact on prevention than on treatment.

A gauge of the impact on drastic weight reduction on cancer risk is given by studies on patients subjected to bariatric surgery. A recent review/meta-analysis concluded that about a 50% reduction in the risk of developing cancer is observed after bariatric surgery in obese patients [100].

Bariatric surgery is obviously a rather dramatic way to reduce weight in a population of subjects at extremely high risk for cancer development, which may explain the magnitude of cancer risk reduction. To what extent can intentional weight loss in less obese/overweight subjects lead to reduced cancer risk? If mutations are accumulated as a function of time and if this process can be hastened or slowed down by systemic metabolism, then it appears evident that a person who has experienced 40 years of obesity is not going to immediately shift his/her cancer risk to that of a normal weight person with a fast weight reduction, however sustained. A telling nation-wide experiment was involuntarily carried out in Cuba: economic crisis between 1990 and 1995 translated into an abrupt decline of obesity prevalence from 15–7%, which went back to pre-crisis periods by year 2003, to rise to almost 20% in 2010. Although incidence and mortality for diabetes and coronary artery disease decreased in parallel with obesity decline, cancer rates remained roughly the same, suggesting at least a significant delay between weight loss and cancer risk reduction [101].

However, there is also evidence for a significant decline in cancer mortality upon intentional weight loss, although trials so far have been too heterogeneous in terms of design and endpoints [27]. There is growing interest in the introduction of lifestyle intervention plans in the therapeutic setting, mostly during adjuvant therapy. Studies in this sense are intrinsically difficult to carry out, and most of the existing literature is comprised of feasibility studies with short follow-up and with non-oncological endpoints, like weight reduction and normalisation of metabolic parameters. The two main studies analysing oncological outcome after lifestyle intervention were the Women’s Healthy Eating and Living (WHEL) [102], and the Women’s Intervention Study (WINS) [103]. The results were dramatically different: WHEL showed no outcome difference between the two arms, whereas in WINS the experimental arm showed an important (24%) but poorly statistically significant (p=0.034) reduction of relapse. Thus, when in front of an obese cancer patient, the clinician still has little evidence to inform his decisions. Should the patient be put on a lifestyle-changing programme, and if yes which? Or would this be unfeasible, useless, or even harmful? Can we predict which patients and which at-risk subjects are likeliest to benefit?

Differences between WINS and WHEL also highlight an important point that deserves to be stressed. There are several ways through which one could decide to achieve and maintain a lower weight. Reducing calorie intake is the main factor to achieve weight loss, no matter how individual nutrients are balanced in the diet [104]. But it is exceedingly difficult to tease apart the effect of excess (or decrease thereof) of specific nutrients on cancer mortality. Is it sufficient to just eat less and maintain ideal weight (hence, total calories are the crucial factor), or does nutrient balance matter? Although neither WINS nor WHEL posed weight loss as an endpoint, their intervention plans (promoting more fiber in WHEL and less fat in WINS) resulted in important differences in weight progression: WHEL patients maintained their calorie intake and moderately gained weight, whereas WINS patients reduced calorie intake by 10% and lost 1.1 BMI points. Thus, weight loss through calorie restriction was associated with improved outcome. A vegetarian diet (independent on its ability to reduce calorie intake) might be more beneficial in preventing onset rather than relapse, although the results of two large prospective trials on colon cancer are strongly conflicting: the recent Adventist Health Study 2 showed a clear risk reduction [105], whereas the EPIC-Oxford showed no difference, after an initial paradoxical increase in risk that was probably due by chance after too early follow-ups [106, 107].

Lifestyle-changing interventions should be sustainable in the long term by cancer survivors, a population affected by significant psychological stress in which long-term adherence to lifestyle recommendations is low.

Finally, too little weight has been given on differences in tumour biology. In contemporary clinical practice, molecular features influence outcome and dictate treatment choice; analogously, they may influence susceptibility to systemic metabolism, as suggested by xenograft studies demonstrating that PIK3CA or PTEN mutant cancers are not susceptible to therapeutic calorie restriction [108]. In the WINS trial, there was a trend for a more elevated benefit from diet in ER-negative patients.

Currently, four main randomised trials (reviewed in [109]) are testing the impact of lifestyle change programmes against a control arm in which generic, non-supervised lifestyle recommendations are administered, using primary oncological endpoints (rate of cancer recurrence, DFS): the Italian DIANA5, the German SUCCESS-C, and the North-American LISA and ENERGY trials. These studies are likely to bring about highly sought after evidence for grounding clinical decisions.

## Conclusion

The discourse on obesity and cancer has become dominant in public debate. Some aspects of this issue are clearly established beyond any reasonable doubt, namely the impact of obesity prior to diagnosis on specific cancer risks and on survivor outcome. Other aspects, such as the exact molecular mechanisms and the extent to which lifestyle changes can modify risk, still await definitive proof. Ultimately, the debate revolves on how much we can modify our cancer risk by intervening on the most basic of animal activities: nutrition. Some might find it tempting to dismiss this debate on the grounds that cancer risk (especially for those cancers where a single environmental factor cannot be identified) is mostly determined by ‘bad luck’, an un-escapable randomness in our stem cells’ DNA mutation accumulation rate [52]. It is clearly not so, but it is also true that our understanding of how nutrition impacts on cancer is far from complete. This creates fertile ground for the proliferation of pseudo-scientific recommendations and bogus ‘super healthy’ nutritional supplements. Likely, some mechanisms and specific lifestyle interventions may have stronger impact on specific cancers. It is also likely that an individual’s genetic makeup may influence response to diet, as we find variable genetic predisposition to specific cancers and genetically determined differences in metabolism. In the wave of medicine personalisation, it is not hard to imagine a future in which lifestyle plans will be tailored to individual risk profile and metabolism.

## Figures and Tables

**Table 1.** Mazzarella

Study abbreviation	Study name	Link	Brief description	Link	Brief description
**USA**					
NIH-AARP	National Institutes of Health-American Association of Retired Persons	http://dietandhealth.cancer.gov/	mail-based questionnaire obtained from 500.000 retired people aged 50-71	http://dietandhealth.cancer.gov/	mail-based questionnaire obtained from 500.000 retired people aged 50-72
CPS-II	Cancer Prevention Study II	http://www.cancer.org/research/researchtopreventcancer/currentcancerpreventionstudies/cancer-prevention-study	mortality study on 1.2 million American men and women. Additional subset with nutritional focus	http://www.cancer.org/research/researchtopreventcancer/currentcancerpreventionstudies/cancer-preventionstudy	mortality study on 1.2 million American men and women. Additional subset with nutritional focus
WHI	Women’s Health Initiative	https://www.nhlbi.nih.gov/whi/	161,808 women aged 50-79 from 40 Clinical Centres. Also randomised dietary intervention trial on a subset	https://www.nhlbi.nih.gov/whi/	161,808 women aged 50-79 from 40 Clinical Centres. Also randomised dietary intervention trial on a subset
NHS II	Nurses Health Study II	http://www.channing.harvard.edu/nhs/	Mail based survey of 116,686 nurses	http://www.channing.harvard.edu/nhs/	Mail based survey of 116,686 nurses
**Europe**					
ORDET	hormones and diet in the aetiology of breast tumours	http://www.ncbi.nlm.nih.gov/pubmed/8614008	10,786 healthy women 35-69 resident in Northern Italy	http://www.ncbi.nlm.nih.gov/pubmed/8614009	10,786 healthy women 35-69 resident in Northern Italy
EPIC	European Prospective Investigation into Cancer and Nutrition	http://epic.iarc.fr/	521 000 participants recruited across 10 European countries. Several smaller cohorts to assess specific endpoints	http://epic.iarc.fr/	522 000 participants recruited across 10 European countries. Several smaller cohorts to assess specific endpoints
Bhaskaran et al	Bhaskaran et al Lancet 2014	http://www.thelancet.com/journals/lancet/article/PIIS0140-6736%2814%2960892-8/abstract	Analysis of data from 5.24 million British Individuals in the Clinical Practice Research Datalink	http://www.thelancet.com/journals/lancet/article/PIIS0140-6736%2814%2960892-8/abstract	Analysis of data from 5.24 million British Individuals in the Clinical Practice Research Datalink
